# A Lupin (*Lupinus angustifolius*) Protein Hydrolysate Decreases the Severity of Experimental Autoimmune Encephalomyelitis: A Preliminary Study

**DOI:** 10.3390/ijms26010032

**Published:** 2024-12-24

**Authors:** Ivan Cruz-Chamorro, Ana Isabel Álvarez-López, Guillermo Santos-Sánchez, Nuria Álvarez-Sánchez, Justo Pedroche, María del Carmen Millán-Linares, Patricia Judith Lardone, Antonio Carrillo-Vico

**Affiliations:** 1 Instituto de Biomedicina de Sevilla, IBiS/Hospital Universitario Virgen del Rocío/CSIC/Universidad de Sevilla, 41013 Seville, Spain; aialvarez-ibis@us.es (A.I.Á.-L.); gsantos-ibis@us.es (G.S.-S.); plardone@us.es (P.J.L.); 2 Departamento de Bioquímica Médica y Biología Molecular e Inmunología, Facultad de Medicina, Universidad de Sevilla, 41009 Seville, Spain; nalvarez-ibis@us.es; 3 Department of Food & Health, Instituto de la Grasa, CSIC, Ctra, Utrera Km 1, 41013 Seville, Spain; j.pedroche@csic.es (J.P.); mcmillan@ig.csic.es (M.d.C.M.-L.)

**Keywords:** hydrolysates, vegetable, neurodegeneration, functional foods, EAE, MS

## Abstract

Multiple sclerosis (MS) is a neurodegenerative disease, with inflammation and oxidative stress in the central nervous system being the main triggers. There are many drugs that reduce the clinical signs of MS, but none of them cure the disease. Food proteins have been shown to contain encrypted peptides that can be released after hydrolysis and exert numerous biological activities. Recently, we have demonstrated the anti-inflammatory and antioxidant activities of a lupin protein hydrolysate (LPH) both in vitro and in vivo. Therefore, the aim of this study was to evaluate whether LPH is capable of reducing the clinical signs of experimental autoimmune encephalomyelitis (EAE), a mouse model of MS. EAE was induced in female C57BL/6N mice and they were treated intragastrically with LPH (100 mg/kg) or vehicle (control group) from day 0 (prophylactic approach) or from the onset of the disease (day 12 post-induction; therapeutic approach) and the clinical score of each mouse was recorded daily. Prophylactic treatment with LPH reduced the clinical score of the mice compared to the control group, as well as the maximum and cumulative scores, without changing the day of onset of the symptoms while the therapeutic intervention did not significantly improve the severity of the disease. For the first time, we demonstrated that prophylactic administration of LPH reduces the severity of MS, suggesting a potential nutraceutical or new functional foods in neuroinflammation. However, further studies are needed to confirm this nutritional effect in a clinical context.

## 1. Introduction

Multiple sclerosis (MS) is a demyelinating disease of the central nervous system (CNS) that causes diffuse neurodegeneration [1]. It currently affects 2.8 million people who suffer from this disease worldwide [2]. MS is considered a multifactorial disease [3] and, although its aetiology is unknown, inflammation [4] and oxidative stress [5] play an important role in the pathophysiology [6]. The most common clinical form of MS (80–90% of patients) is relapsing-remitting MS (RR-MS), which is characterised by alternating periods of disease and recovery [7]. The study of MS pathogenesis is complicated because it is difficult to examine brain tissue damaged during active disease. Therefore, most studies are carried out in the preclinical model of MS in mice, called experimental autoimmune encephalomyelitis (EAE). EAE follows a predictable clinical course, characterised by a prodrome period of about 10–12 days, followed by ascending paralysis from the tail and hind limbs, progressing to the forelimbs, along with weight loss [8,9]. After inoculation with myelin oligodendrocyte glycoprotein 35–55 (MOG_35–55_), autoreactive immune cells, once activated in secondary lymphoid tissue, circulate through the blood until they reach the CNS. Here, they are reactivated by resident antigen-presenting cells and induce a massive release of immune mediators and reactive oxygen species (ROS), among others, which trigger demyelination and the subsequent neurodegeneration, leading to paralysis of the animals [10]. Currently, there is no cure for MS and most approved drugs are immunomodulators that counteract the excessive immune response and are able to control the progression of disability and the number of relapses in patients [11].

In the last two decades, the field of functional foods based on food-derived peptides has attracted the attention of the scientific community due to their numerous health benefits [12]. Although these are primarily focused on the prevention and treatment of hypertension, other food-derived peptides (wheat, shrimp, milk, pork, hempseed, or rice) are currently in a preclinical stage or in clinical trials for the treatment of other non-communicable diseases (including neurological diseases) [13]. To date, numerous food-derived peptides from whey, fish, maize, rice, walnut and others, have been shown to exert anti-inflammatory and antioxidant effects in PC12 (neuroendocrine), SH-SY5Y (neuroblastoma), and BV-2 (microglial) cell lines [14]. Other peptides have been shown to improve memory, learning, antioxidant and anti-inflammatory status, and neuroprotection in the CNS in in vivo models [14]. However, no previous study has evaluated the effect of food-derived peptides on the clinical signs of EAE.

In our previous work, we have shown that a hydrolysate obtained with Alcalase^®^ of *Lupinus angustifolius* seed proteins (named LPH), exerts antioxidant and immunomodulatory effects in vitro, in mouse models, and in a clinical food trial (Lupine-1). Furthermore, we have recently demonstrated the anxiolytic-like effects of LPH in an anxious mouse model. In addition, we have shown for the first time that LPH improves the antioxidant status of the CNS, which may be a possible explanation for the observed anxiolytic effect (revised in [15]).

In light of these considerations, and because oxidative and inflammatory damage is one of the main factors associated with neurodegenerative diseases, this study aimed to evaluate the effects of LPH on the clinical signs of EAE, a mouse model of multiple sclerosis.

## 2. Results

### 2.1. Prophylactic LPH Treatment Reduces the Clinical Severity of EAE

**Figure 1:**
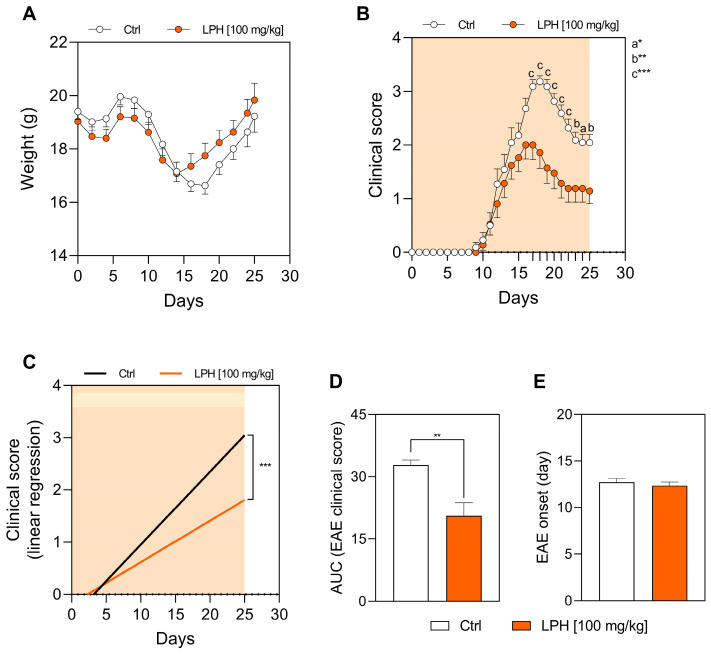
Weight of mice and clinical EAE score in the prophylactic approach. (**A**) Weight of animals during the experiments. (**B**) Clinical EAE score. (**C**) Linear regression of the clinical EAE score. (**D**) AUC of the clinical EAE score. (**E**) Day of onset of the clinical signs manifestation. * *p* ≤ 0.05; ** *p* ≤ 0.01; *** *p* ≤ 0.005; *n* = 20. AUC, area under the curve; Ctrl, control group; EAE, experimental autoimmune encephalomyelitis; LPH, lupin protein hydrolysate. The orange shade shows the duration of the treatment.

The two experimental groups showed normal disease-related weight fluctuations throughout the course of EAE, but no significant differences were observed between them (Figure 1A).

Regarding the clinical signs of the disease, the control group reached the peak of the disease at day 18 p.i., with a mean EAE score of 3.2 ± 0.11 (Figure 1B, white dots). Treatment with LPH significantly reduced the clinical signs of EAE (Figure 1B, orange dots) from the day 17 p.i. onwards. In fact, on day 18 p.i., LPH-treated mice showed a mean EAE score of 1.9 ± 0.29 (*p* = 0.0003 vs. Ctrl group). Two videos are provided in the Supplementary Materials for further visual appreciation. A representative vehicle-treated mouse at 18 days p.i. of EAE was recorded in Supplementary Video S1, showing a clinical sign score = 3, i.e., paralysis of one hind limb (in this case the left limb). Meanwhile, a representative LPH-treated mouse at 18 days p.i. of EAE was recorded in Supplementary Video S2, showing a clinical sign score = 2, i.e., impaired righting reflex. Linear regression of the EAE score curve (Figure 1C) and area under the curve (AUC) calculation (Figure 1D), showed the significant beneficial effect of the LPH treatment. In fact, the slope value obtained from the linear regression of the Ctrl group was 0.134, while the LPH group showed a value of 0.079 (*p* < 0.005) (Figure 1C). The AUC calculated for the Ctrl group was 32.80 ± 2.52, while for the LPH group was 20.57 ± 3.26 (*p* < 0.005) (Figure 1D). LPH treatment did not alter the day of onset of the clinical signs, compared to vehicle-treated mice (Figure 1E).

**Figure 2:**
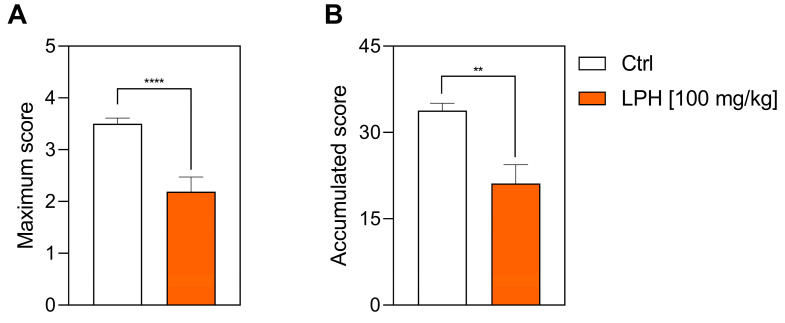
Severity of the EAE in the prophylactic approach. (A) Maximum score and (B) accumulated score reached by the two experimental groups. ** *p* ≤ 0.01; **** *p* ≤ 0.0001; *n* = 20. Ctrl, control group; LPH, lupin protein hydrolysate.

Although the day of onset of clinical signs was not different between the two experimental groups, LPH-treated mice showed a mean maximum score of 2.2 ± 0.28 (Figure 2A), which was lower than the Ctrl group (3.5 ± 0.11) with a strong statistical difference (*p*-value < 0.0001).

Finally, the sum of all the daily scores (i.e., accumulated score) was analysed. As shown in Figure 2B, the treatment with LPH decreased the total cumulative score compared to vehicle-treated mice (Ctrl: 33.82 ± 1.26 vs. LPH: 21.14 ± 3.23, *p*-value = 0.006).

### 2.2. Therapeutic LPH Treatment Does Not Ameliorate Ongoing EAE

To test whether LPH could reverse the disease once it had started, mice were treated with LPH from the onset of clinical signs (considered to be around day 12 p.i. in the previous prophylactic experiments) until the end of the experiment (day 25 p.i.).

**Figure 3:**
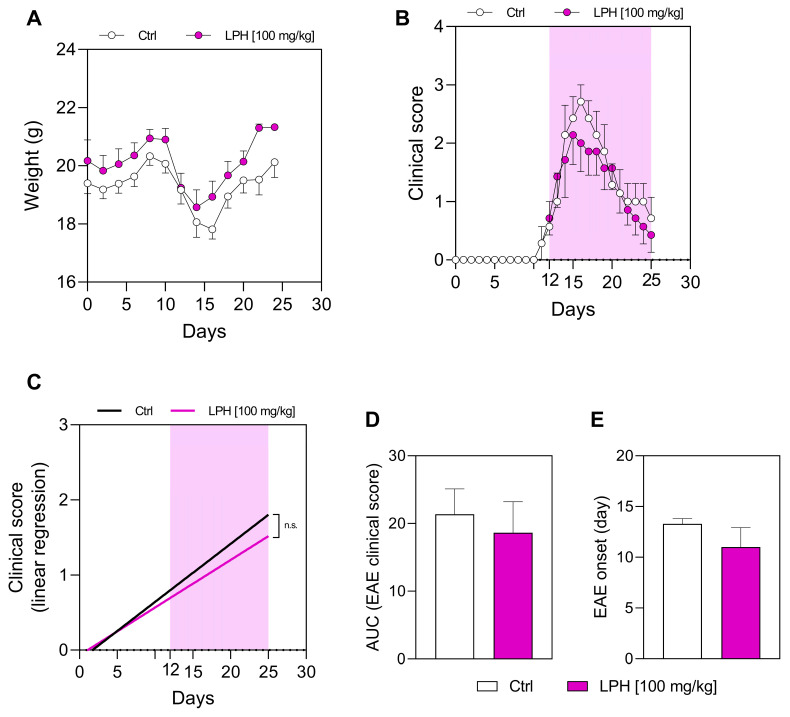
Weight of mice and clinical EAE score in the therapeutic approach. (**A**) Weight of animals during the experiments. (**B**) Clinical EAE score. (**C**) Linear regression of the clinical EAE score. (**D**) AUC of the clinical EAE score. (**E**) Day of onset of the clinical signs manifestation. *n* = 8. AUC, area under the curve; Ctrl, control group; EAE, experimental autoimmune encephalomyelitis; LPH, lupin protein hydrolysate; n.s., not significant. The purple shade shows the duration of treatment.

Throughout the therapeutic experiments, the body weight of the animals (Figure 3A), as well as the EAE score curve of the Ctrl group (Figure 3B, white dots), were similar to the prophylactic experiments (Figure 1A,B, respectively). However, LPH treatment was unable to reduce the clinical signs of EAE (Figure 3B, purple dots). Thus, there were no significant differences in the linear regression of the EAE score (Figure 3C) or the AUC data (Figure 3D) between the two experimental groups. In fact, the slope value of the Ctrl and LPH groups was 0.077 and 0.063, respectively, with a *p*-value of 0.309 (Figure 3C); while the values obtained by the calculation of AUC were 21.36 ± 2.70 and 18.64 ± 2.87 for the Ctrl and LPH groups, respectively (Figure 3D), without reaching statistical significance (*p* = 0.778).

The mean of the day of EAE onset was maintained around day 12 p.i. for both experimental groups, without significant differences (Figure 3E), and the severity of the disease was unchanged between the two groups.

**Figure 4:**
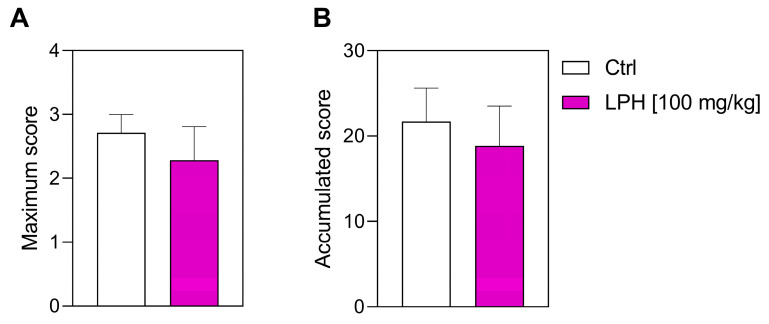
Severity of the EAE in the therapeutic approach. (**A**) Maximum score and (**B**) accumulated score reached by the two experimental groups. *n* = 8. Ctrl, control group; LPH, lupin protein hydrolysate.

Treatment with LPH did not decrease the maximum score achieved by the mice (2.3 ± 0.5), compared to the Ctrl group (2.7 ± 0.3, *p*-value = 0.776) (Figure 4A) and the analysis of the accumulated score was not modified by LPH treatment compared to the Ctrl group (Ctrl: 21.71 ±3.90 vs. LPH: 18.86 ± 4.65, *p*-value = 0.778) (Figure 4B).

### 2.3. LPH Has Biologically Active Peptides

**Figure 5:**
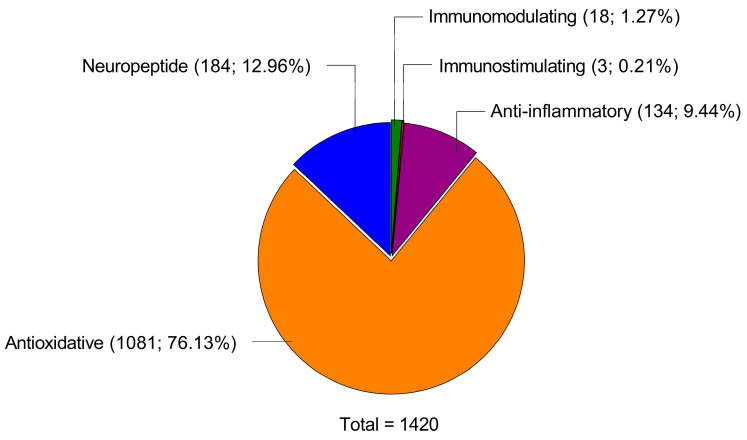
Pleiotropic effects exerted by the LPH sequences. Number and percentage of the lupin protein hydrolysate sequences that contain a described motif with the biological effect.

LPH sequences were analysed for the presence of motifs (short amino acid sequences) with proven biological activity. In a previous study, a total of 1685 sequences were identified in LPH [16]. Of these, 1420 sequences contained at least one motif with a previously demonstrated biological effect, such as antioxidant (1081), neuropeptide (184), anti-inflammatory (134), immunomodulatory (18), and immunostimulant (3) activities (Figure 5). Detailed motifs contained in these sequences are reported in Table 1.

## 3. Discussion

In this study, we have demonstrated for the first time that prophylactic administration of LPH at 100 mg/kg to EAE mice reduces the severity of the disease. The pathogenesis of MS has been related to altered redox homeostasis, and several authors have suggested that this phenomenon is not limited to the CNS, but may also occur in the periphery in patients with MS [18]. High levels of ROS damage the brain endothelium and increase the permeability of the blood–brain barrier (BBB), facilitating the entry of immune cells into the brain parenchyma [19,20]. In fact, one of the key points in the development of MS is the migration of immune cells into the CNS by crossing the BBB. Moreover, excessive ROS production has been directly identified as a mediator of demyelination and axonal damage in both MS and EAE [21,22]. In this context, several reports have shown that the therapeutic manipulation of oxidative stress mechanisms ameliorates the clinical signs of MS. Ruuls et al. (1995) demonstrated that intraperitoneal administration of catalase (CAT) prior to the onset of neurological deficit delayed the onset of EAE and reduced its duration and severity [23]. Moreover, the synthetic complex with combined superoxide dismutase (SOD) and CAT activity ameliorated EAE and allowed full recovery of the mice after 40 days [24]. Our previous studies have shown that LPH increased the SOD and CAT activities and total antioxidant capacity (TAC) both in in vitro cultured human lymphocytes [25] and in the plasma of hypercholesterolemic mice [26], demonstrating its efficacy and non-toxic effect. In addition, LPH alleviated the high-fat diet-induced decrease in total antioxidant activity in the CNS by increasing cerebral glutathione levels and the activity of the antioxidant enzymes CAT and GR, resulting in decreased levels of 8-hydroxy-2′-deoxyguanosine levels, a marker of DNA oxidative damage [27]. On the other hand, MS is characterised by excessive production of pro-inflammatory cytokines by immune cells infiltrating the CNS [28]. In particular, effector Th1 CD4+ T cells, represented by the production of tumour necrosis factor (TNF) and interferon (IFN)-γ cytokines, play a pathological role in neuroinflammatory responses associated with EAE and MS, while regulatory responses (Th2 and Treg) suppress excessive inflammation in MS and EAE, where interleukin (IL)-10 has been shown to be one of the major anti-inflammatory cytokines associated with protection in the context of MS and EAE [29,30]. Along with increased levels of pro-inflammatory cytokines from Th1 cells, polarisation of macrophages and microglia towards the pathogenic profile of M1 (pro-inflammatory) has also been observed during the acute phase in EAE mice [31]. In fact, the use of Fasudil, a drug capable of inducing macrophage polarisation towards the M2 (anti-inflammatory) phenotype reduced the clinical severity of EAE [32]. We have previously reported that LPH favours the production of anti-inflammatory cytokines, such as IL-10 [25], and promotes an anti-inflammatory response by decreasing the Th1/Th2 ratio [25,26]. Additionally, an LPH-isolated peptide has been shown to promote macrophage differentiation towards a protective M2 phenotype and to prevent inflammation in microglial cells, conferring neuroprotection in the brain [15]. In detail, the study conducted on lipopolysaccharide-stimulated BV2 microglial cells demonstrates that treatment with an LPH-derived peptide exerts anti-inflammatory effects by lowering mRNA expression levels of IL-1β, IL-6, and TNF while increasing IL-10 gene expression. Furthermore, this LPH-derived peptide reduced the expression of M1 phenotype markers and increased the expression of M2 phenotype markers. Consequently, this peptide promotes M2 polarisation, helping to prevent prolonged microglial activation, which can contribute to the onset of neurodegenerative diseases [15]. Finally, LPH demonstrated similar effects in an animal model that was fed a Western diet, confirming the shift towards the protective M2-microglia phenotype [15]. Moreover, in a previous study, we demonstrated that LPH treatment reduces aortic immune cell infiltration in a mouse model of hypercholesterolemia by decreasing the levels of some chemokines involved in immune cell recruitment [26]. Thus, chemokine reduction may also contribute to the reduction of the pathogenic cell infiltration in the CNS.

Besides the effects that LPH may exert at the peripheral level by controlling oxidative stress processes and the inflammatory response in lymph nodes in the early stages of EAE, they may also exert their direct effect on the CNS through their ability to cross the BBB [27]. Although the exact mechanism by which peptides cross the BBB remains unclear, previous research suggests that this may occur through processes such as receptor-mediated transcytosis or specific transporters. Examples include the peptide transporter, the large neutral amino acid transporter, and the peptide histidine transporter [33]. Based on this understanding, it will be crucial for future research to focus on elucidating these pathways in more detail, as passage through the BBB is crucial for the treatment of CNS diseases. Exploring other potential mechanisms or unknown transporters that may facilitate peptides passage across the BBB could provide new insights into therapeutic approaches targeting the CNS.

In agreement with the previous results, the chemical analysis of the LPH sequences reveals the presence of several motifs with proven biological activity. In fact, more than 84% of the sequences contain a bioactive motif. Specifically, 76.13% of the sequences contain an antioxidant motif, while 23.87% contain an anti-inflammatory motif. Therefore, the present results support the protective role of prophylactic administration of LPH in EAE.

The bioactivity of a single peptide is generally influenced by its size, physicochemical characteristics, and amino acid sequence. In contrast, the bioactivity of a protein hydrolysate is determined by its overall composition, which includes both active and inactive components, as well as potential synergistic or antagonistic interactions. The composition results from the specific protein source and from the specific hydrolysis conditions, which are influenced by factors such as the type of digestive enzyme or enzyme combination used, as well as temperature, pH, and other parameters. Therefore, the establishment of a clear structure–function relationship in this context is an interesting challenge. Another factor lies in the fact that the structure of peptides can change during digestion and absorption processes [34], as well as, peptides can be modified by enzymes that are in the intestinal lumen [35]. Recently, nanotechnology has emerged as a promising strategy in the food industry to address several limiting factors. The use of this technology to create nano-nutraceuticals can enhance the beneficial properties of nutrients by delivering them in nanostructured forms, such as nanoparticles, nanoemulsions, nanogels, and similar formats (revised in [36]).

This work also shows, for the first time, that LPH treatment in EAE animals, when administered daily starting from the onset of symptoms (around day 12 p.i.), is not capable of reducing the severity of the disease, demonstrating its low efficacy as a therapeutic approach. This finding is consistent because the onset of symptoms in the animal is preceded by advanced axonal damage due to previous neuroinflammatory and oxidative episodes. Thus, starting treatment on day 0 (prophylactic approach) allows LPH to act on immune cells before they enter the CNS.

Unlike the animal model, which simulates a single relapse of the MS, patients with RR-MS suffer from more than one relapse [9]. Therefore, regular consumption of LPH used as a nutraceutical or integrated into the diet as a functional food could contribute to protection in ongoing MS, controlling further exacerbation and reducing disease severity. Undoubtedly, additional studies in patients with RR-MS are needed to confirm this promising effect of LPH on the disease. It would also be interesting to evaluate the effects of co-treatment with LPH and standard MS therapies. In this regard, the concomitant use of LPH with disease-modifying therapies or immunosuppressants may have a synergistic effect, potentially improving therapeutic outcomes, as has recently been shown with other natural compounds and methylprednisolone therapy [37].

Food-derived peptides could have significant potential in the treatment of neurodegenerative diseases. As research in this field advances, the exploration of their biological activities, such as their neuroprotective and regulatory effects on cellular processes, highlights their promise as innovative therapeutic agents. The capability of these peptides to provide a natural and potentially safer alternative to traditional treatments makes them an attractive option for future therapeutic strategies.

One of the remarkable strengths of the LPH evaluated in this study is their multifunctional nature (revised in [15]), which may be particularly beneficial in addressing the complex and multifactorial pathophysiology of MS. Unlike therapies that target single molecules or pathways, LPH has a broad spectrum of effects, including anti-inflammatory and antioxidant properties. Given the unclear and multifaceted aetiology of MS—in which oxidative stress, immune dysregulation, and other environmental and genetic factors interact—this pleiotropic mechanism may contribute to the observed therapeutic effects. It is plausible that LPH’s ability to modulate multiple pathways simultaneously makes it particularly suited to diseases such as MS, where no single factor is exclusively responsible for pathology.

However, despite these promising findings, this preliminary study has several limitations that warrant further investigation. While the clinical outcomes suggest therapeutic potential, they do not provide direct mechanistic insights into how LPH exerts its effects. Key pathological processes such as oxidative stress, inflammation, and demyelination in the CNS were not assessed. Future research should include detailed histopathological analyses to evaluate white matter demyelination, immune cell infiltration, and neuronal preservation in the CNS. Additionally, measurement of cytokine production levels and oxidative stress markers, such as lipid peroxidation and antioxidant enzyme activity, could help to elucidate the pathways modulated by LPH.

The lack of a robust theoretical framework for MS due to its poorly understood aetiology presents both a challenge and an opportunity. The ability of LPH to act on multiple targets could be a critical advantage in the development of therapies for MS and other multifactorial diseases. Advanced methods, such as single-cell transcriptomics and in vivo imaging, could provide deeper insights into the specific cellular and molecular pathways affected by LPH. Such approaches would allow a more comprehensive understanding of their pleiotropic effects and their potential to modulate the multifactorial mechanisms driving MS pathology.

In conclusion, the multifunctional properties of LPH may fit well with the multifactorial nature of MS, offering a promising avenue for therapeutic innovation. Further research is essential to validate their clinical efficacy and mechanistic effects, ultimately paving the way for their translation into effective treatments for neurodegenerative diseases.

## 4. Materials and Methods

### 4.1. LPH Preparation

*Lupinus angustifolius* protein isolate (LPI) was obtained as follows: defatted lupin flour was extracted with 0.25% Na_2_SO_3_ (*w*/*v*) at pH 10.5 for 1 h. After centrifugation, the supernatant was collected, and the pellet was subjected to a second extraction. Both supernatants were adjusted to the isoelectric point of lupin proteins (pH 4.3). The resulting precipitate was washed with distilled water at pH 4.3 and centrifuged to remove residual salts and non-protein compounds. Finally, the precipitated proteins were lyophilised and resuspended in distilled water (10% *w*/*v*). LPI was used to obtain LPH, as described elsewhere [25]. Briefly, the enzyme Alcalase^®^ (Novozymes, Bagsvaerd, Denmark) was used to hydrolyse a LPI for 15 min. At the end of the hydrolysis, the Alcalase^®^ was heat-inactivated, and the solution was centrifuged to obtain the LPH (the supernatant portion). This was lyophilised and dissolved in a saline solution, then filtered, autoclaved, aliquoted, and stored at −80 °C. The chemical and amino acid composition of LPH has previously been reported [26].

### 4.2. Animals and EAE Induction

**Figure 6:**
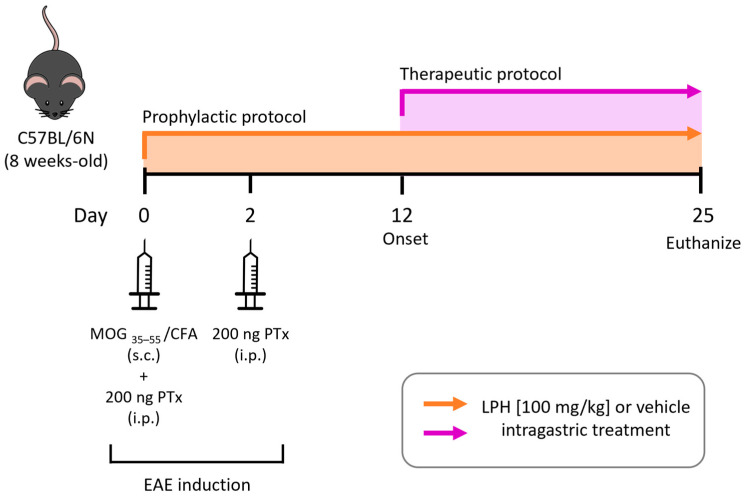
Experimental design and timeline. Schematic diagram of the experimental design of the study. CFA, complete Freud adjuvant; EAE, experimental autoimmune encephalomyelitis; i.p., intraperitoneal injection; LPH, lupin protein hydrolysate; MOG, myelin oligodendrocyte glycoprotein; PTx, pertussis toxin; s.c., subcutaneous injection.

Fifty-six female C57BL/6N mice (8 weeks old) from the University of Seville Animal Facility were housed under standard conditions with ad libitum access to water and food. For the induction of EAE, the previously described protocol was followed [29]. Briefly, 100 µg of MOG_35–55_ peptide (Cambridge Research Biochemicals, Cambridge, UK) emulsified in complete Freud’s adjuvant (CFA, Sigma-Aldrich, St. Louis, MO, USA) containing 4 mg/mL of heat-inactivated *Mycobacterium tuberculosis* (Sigma-Aldrich) was inoculated subcutaneously. Furthermore, two doses of 400 ng pertussis toxin (Enzo Life Sciences, Farmingdale, NY, USA) were administered intraperitoneally on days 0 and 2 post-induction (p.i.) to induce the disease (Figure 6).

An EAE clinical sign score was assigned daily to each mouse as follows: 0 = no clinical sign; 1 = no tail tone; 2 = impaired righting reflex; 3 = paralysis of one hind limb; 4 = paralysis of both hind limbs; 5 = paralysis of both hind limbs and one forelimb; 6 = if the score was greater than 5 (including moribund or dead mouse).

*Prophylactic protocol*: Four separate experiments were carried out. In each experiment, 10 mice were divided into two experimental groups: the mice were treated intragastrically either with vehicle (Control group, Ctrl, *n* = 5) or 100 mg/kg of LPH (LPH group, LPH, *n* = 5) daily on day 0.

*Therapeutic protocol*: Two separate experiments were performed. In each experiment, 8 mice were divided into two experimental groups and intragastric treatment was started at the onset of clinical signs of EAE (~day 12 p.i.). Thus, mice received vehicle (*n* = 4) or LPH (100 mg/kg, *n* = 4) daily until the end of the experiment.

The LPH dose was determined from prior internal experiments and did not demonstrate cytotoxic effects. Based on the guidelines from Reagan-Shaw [38], the corresponding human dose was estimated to be 8.12 mg/kg. Mice were euthanised on day 25 p.i. by CO_2_ inhalation.

All procedures were performed in accordance with European Directive 2010/63/EU, Spanish legislation on animal experimentation (Royal Decree 53/2013) and approved by the Ethics Committee of the Virgen Macarena and Virgen del Rocío University Hospitals (reference 08/05/2024/055).

### 4.3. LPH Peptides Identification

LPH sequencing was performed as previously described using nano high-performance liquid chromatography coupled with an Orbitrap Elite mass spectrometer [16]. The BIOPEP-UWM database was used to identify the motifs with proven biological activity [17].

### 4.4. Statistical Analysis

Data were presented as mean ± standard error of the mean (SEM). Data were analysed using either the Mann–Whitney U test or two-way ANOVA with post hoc correction using GraphPad Prism 8 (GraphPad Software, San Diego, CA, USA). Differences with a *p*-value ≤ 0.05 were considered statistically significant.

## 5. Conclusions

This study demonstrates for the first time that a lupin protein hydrolysate produced by the enzyme Alcalase^®^ reduces the severity of EAE and may be a potential source of nutraceuticals in MS. Although LPH treatment (100 mg/kg) administered at the onset of the clinical signs of EAE does not improve the severity of the disease, daily prophylactic treatment ameliorated the clinical symptoms controlling the evolution of the disease. Therefore, LPH could be pointed out as a nutraceutical with a potential clinical impact. However, further investigation of the molecular mechanisms in the CNS and clinical trials should be carried out to elucidate the effect of LPH on MS.

## Figures and Tables

**Table 1:** Peptide motifs content in the LPH sequences with biological activity.

Biological Activity	Number of Sequences	Peptide Motive ^a^
Immunomodulating	7	KRP
	4	RKP
	4	YKPR
	1	GRKP
	1	TKRP
	1	YGG
Total sequences	18	
Immunostimulating	1	GFL
	1	EAE
	1	LGY
Total sequences	3	
Anti-inflammatory	87	PY
	23	HY
	15	VPP
	6	IPP
	2	YW
	1	ANP
Total sequences	134	
Antioxidative	101	KP
	98	IR
	89	LK
	67	KD
	64	EL
	43	RY
	42	RHR
	37	LY
	37	KVI
	29	PYY
	29	LLPH
	29	AY
	27	HL
	26	YYF
	25	HH
	23	LH
	21	IY
	16	PHY
	13	VY
	12	YNL
	11	PW
	9	AH
	9	RHG
	9	RW
	9	TW
	8	PEL
	8	LPL
	7	IKK
	7	WY
	7	LHL
	7	RHN
	7	IKL
	7	TY
	6	PHH
	6	HHH
	6	RHE
	6	VKL
	6	EAK
	6	VKP
	6	NEN
	5	VKV
	4	RHQ
	4	RHT
	4	GGE
	4	LKP
	4	VW
	4	LPILR
	3	AHH
	3	HRH
	3	PHL
	3	PHW
	3	RWL
	3	MY
	3	VVKL
	3	LLR
	3	AGDDAPR
	3	SVL
	2	ADF
	2	GHH
	2	HKH
	2	PWA
	2	PWD
	2	PWT
	2	PWW
	2	VYV
	1	IHH
	1	DHH
	1	EHH
	1	LHA
	1	LHR
	1	LWH
	1	PHF
	1	PHI
	1	PHS
	1	PHV
	1	PWG
	1	PWL
	1	PWR
	1	RHF
	1	RWN
	1	RWR
	1	KAI
	1	TDY
	1	AW
	1	LW
	1	GPP
	1	WG
	1	MM
	1	DYK
	1	KKY
	1	KYL
	1	VAPEEHPV
	1	GSH
	1	LGY
	1	RYL
	1	YLG
Total sequences	1081	
Neuropeptide	100	GQ
	42	YL
	18	KPS
	13	YR
	5	KPT
	4	YKPR
	1	YLG
	1	MH
Total sequences	184	
**Total sequences**	**1420**	

^a^ 1-Letter amino acid code; LPH, lupin protein hydrolysate. The BIOPEP-UWM database was used to identify motifs with demonstrated biological activity [17].

## Data Availability

Data are available in a publicly accessible repository.

## Supplementary Materials

The following supporting information can be downloaded at: https://www.mdpi.com/article/10.3390/ijms26010032/s1.
